# Population Structure and Local Adaptation of *Acrossocheilus yunnanensis* in the Headwaters of the Chishui River

**DOI:** 10.3390/ani16142135

**Published:** 2026-07-09

**Authors:** Ji Huang, Xianjie Huang, Qun Lu, Jianhu Liu, Shuang Li, Mengru Wang, Chunlin Zhang

**Affiliations:** 1 College of Fisheries, Southwest University, Chongqing 400715, China; 2 Yunnan Administration and Protection Bureau of Rare and Endemic Fish National Nature Reserve in the Upper Reaches of the Yangtze River, Zhaotong 657900, China

**Keywords:** *Acrossocheilus yunnanensis*, whole-genome resequencing, genetic structure, local adaptation

## Abstract

As a major tributary of the upper Yangtze River, the Chishui River possesses distinctive ecological frameworks and serves as a critical refuge for the aquatic germplasm resources of the Yangtze Basin. *Acrossocheilus yunnanensis*, a dominant native fish endemic to the river’s headwaters, contributes substantially to sustaining regional ecosystem functioning. Nevertheless, no population genetic research on this species has been documented in the headwater reaches of the Chishui River to date. Here, whole-genome resequencing was performed on 140 individuals from seven geographical populations of *A. yunnanensis* sampled from the headwaters of the Chishui River. A total of 242,504,293 high-quality single-nucleotide polymorphism (*SNP*) markers were identified, with an average sequencing depth of 10×. Population genetic analyses revealed observed heterozygosity (H_O_) ranging from 0.290 to 0.340, expected heterozygosity (He) from 0.303 to 0.333, and nucleotide diversity (π) spanning 8.063 × 10^−4^ to 1.086 × 10^−3^. Genome-wide *F_ROH_* values were generally low across the seven populations, and only several individuals within Population A exhibited clear signs of inbreeding risk. Pairwise differentiation results indicated that the Banbanqiao (A) was highly genetically divergent from the other six populations. Further genome-wide selective sweep analyses verified that A has evolved unique local adaptive signatures in pathways related to mechanosensory perception, neuromotor integration, anaerobic metabolic remodeling and protein homeostasis regulation. This study systematically characterizes the population genetic architecture and local adaptation mechanisms of headwater *A. yunnanensis*, providing pivotal genetic evidence for wild germplasm conservation and scientific aquatic ecosystem management across the watershed.

## Simple Summary

*Acrossocheilus yunnanensis* plays an important ecological role in the headwaters of the Chishui River; however, research on its genetic structure and environmental adaptation in this region remains inadequate. Therefore, whole-genome resequencing was applied to analyze the genetic structure of seven geographic populations in this area, and runs of homozygosity (ROH) were further utilized to calculate F_ROH_ for evaluating the genomic health of each population. In addition, this study found that the Banbanqiao (A) population has formed unique adaptive characteristics in mechanosensation, neuromotor integration, anaerobic metabolic remodeling, and related pathways. These findings provide a theoretical framework for the conservation and sustainable management of *A. yunnanensis* germplasm resources in the headwaters of the Chishui River.

## 1. Introduction

As an important first-order tributary on the southern bank of the upper Yangtze River [1], the Chishui River serves as a critical sanctuary for rare and endemic aquatic species. The headwater region of this basin is characterized by deeply incised valleys, fragmented topography, and pronounced spatial habitat heterogeneity. This unique geographic environment has driven the population differentiation and local adaptive evolution of indigenous fish species [2]. As a dominant indigenous fish in the Chishui River headwaters, *Acrossocheilus yunnanensis* primarily feeds on filamentous algae and aquatic mosses [3], playing a vital role in regulating algal community structure and maintaining stream ecosystem balance [4,5,6]. Therefore, the stability of its population is paramount to safeguarding the aquatic biodiversity and ecosystem integrity of the entire basin.

Population genetic diversity serves as a core indicator for evaluating the germplasm health, environmental adaptability and evolutionary potential of species, and lays an essential foundation for species conservation and germplasm assessment. Traditional population genetic studies on fish mostly rely on low-throughput molecular markers such as mitochondrial DNA and SSRs. These markers are limited by small number of markers, low genome coverage and insufficient resolution, making it difficult to accurately resolve fine-scale population genetic structure and key variants linked to environmental adaptation [7,8,9]. In recent years, whole-genome resequencing has developed rapidly. Benefiting from high marker density and full genomic coverage, this technique efficiently identifies massive genetic variants, including SNPs and InDels, and provides high-resolution data to systematically uncover population differentiation, genetic diversity and habitat adaptation mechanisms in fish. Numerous published works have verified its practical value. Yang et al. [10] analyzed the population differentiation of landlocked *Neosalanx brevirostris* in the Yangtze River using whole-genome resequencing. Multiple adaptive variants related to osmoregulation, immune response, locomotion and energy metabolism were screened via selective sweep analysis, revealing its genetic adaptation to specific habitats. Based on resequencing data, Gao et al. [11] found weak overall genetic differentiation and low genetic diversity in *Schizothorax oconnori* from the Yarlung Tsangpo River; only the Zangga population formed an independent genetic lineage. The authors proposed differentiated conservation units and identified selected genes enriched in DNA repair and energy metabolism pathways, confirming the vital roles of these pathways in fish adaptation to high-altitude extreme environments. In summary, whole-genome resequencing enables the accurate detection of subtle population divergence and functional adaptive variants, which is more suitable for refined germplasm evaluation and adaptive evolutionary research in fish.

Current research on *A. yunnanensis* has mainly focused on biological aspects such as growth, reproduction, and immunity [12]. In terms of genetic investigations, Yang et al. [13] analyzed the genetic structure and differentiation of nine populations from multiple rivers, including the Hongshui River, Jinsha River, and Nanpan River, using four mitochondrial gene sequences. The populations were classified into four groups, which preliminarily clarified the overall genetic diversity and phylogenetic relationships of the species. Owing to the limited genetic variation information provided by mitochondrial genes, previous studies have been unable to further resolve the historical evolution and environmental adaptation characteristics of the species. In addition, existing genetic surveys have not extended to the headwater area of the Chishui River, resulting in a lack of systematic exploration of populations in this special watershed. The genetic diversity and population differentiation patterns of *A. yunnanensis* in the headwater region remain to be further explored. Based on the complex habitat characteristics of the headwater section, we speculated that geographical isolation and localized environmental pressures have driven distinct population genetic differentiation and local adaptive evolution in *A. yunnanensis*. To verify this speculation, this study employed the chromosome-level genome assembled in our previous research, combined with whole-genome resequencing technology, to preliminarily analyze the population structure and local adaptive characteristics of *A. yunnanensis* in the headwater section of the Chishui River. It also clarified the genetic relationships and germplasm status among populations from different headwater tributaries. The findings provide important scientific evidence for the conservation of fish resources, watershed ecological restoration, and the development of characteristic aquaculture germplasm resources in the upper Yangtze River.

## 2. Materials and Methods

### 2.1. Sample Collection and DNA Extraction

**Figure 1:**
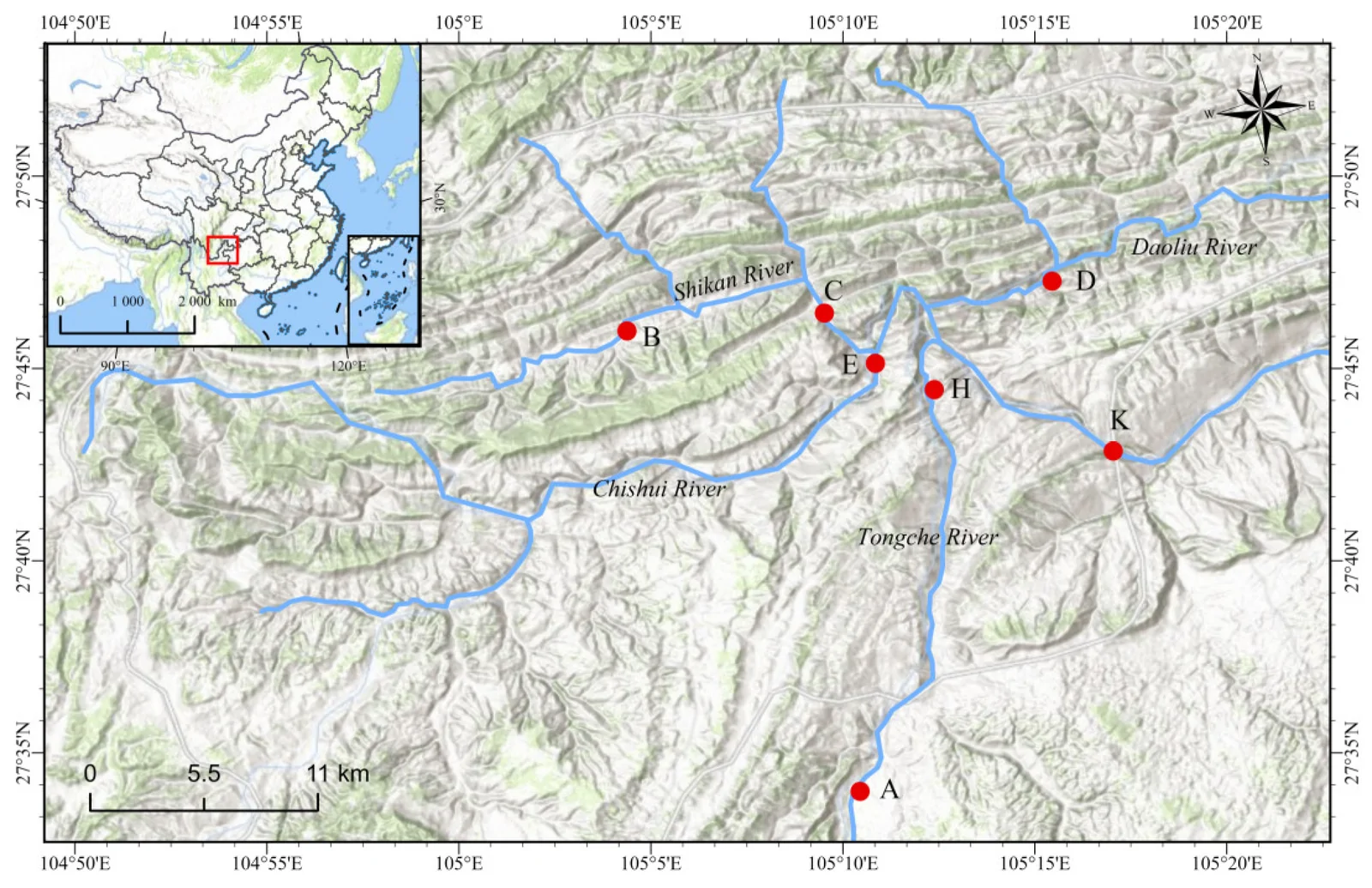
Sampling sites of *A. yunnanensis*. Red dots indicate the sampling locations.

Samples were collected during the fish resource survey conducted in the headwater section of the Chishui River from June to December 2025. Random sampling was carried out along the mainstream and tributaries of the upper Chishui River, including the Tongche River, Daoliu River and Shikan. The sampling area covered all major tributaries in this region, covering upstream and downstream habitats across the main distribution range of adult *A. yunnanensis*. The specific sampling sites included Banbanqiao (A), Dashancun (B), Wuqiutian (C), Wanjiaotang (D), Erlongqiangbao (E), Chenjiawan (H), and Guohaxia (K). To ensure sample representativeness, 20 adult individuals were collected at each site. The detailed sampling locations are shown in Figure 1 and Table 1. During sampling, fin clip tissues (0.1–0.2 g) were taken from each individual and immediately preserved in 95% ethanol for subsequent genomic DNA extraction. A total of 140 qualified individuals were obtained for whole-genome resequencing. Genomic DNA was extracted using the CTAB method. The concentration and purity of DNA were measured with a NanoDrop 2000 spectrophotometer (Thermo Fisher Scientific, Wilmington, DE, USA), and DNA integrity was verified via 1.2% agarose gel electrophoresis. Qualified DNA samples were diluted to 50 ng/μL and stored at −20 °C for further use.

### 2.2. Library Construction and Resequencing

After quality assessment of the genomic DNA, samples were fragmented by mechanical shearing (ultrasonication). Fragments of the desired size were selected, followed by end repair, 3′-end A-tailing, adapter ligation, and PCR amplification and purification. The amplified products were subjected to single-strand circularization to form circular DNA. Using the circular DNA as a template, DNA nanoballs (DNBs) were generated via rolling circle amplification, a unique linear amplification mode. The prepared DNB library was then loaded onto the patterned array sites on the sequencing chip, and 150 bp paired-end sequencing was performed on the BGI DNBSEQ platform according to the manufacturer’s instructions.

### 2.3. Quality Control and Sequence Alignment

Raw data were first processed using fastp software (v0.21.0, parameters: -q 10 -u 50 -y -g -Y 10 -e 20 -l 100 -b 150 -B 150). We removed adapter sequences at both ends of the reads, reads with more than 40% of bases of Phred quality of Q < 15, reads with trailing polyG sequences, reads with sequence complexity lower than 10%, reads shorter than 100 bp, and single-end reads carrying more than five ambiguous N bases to generate high-quality clean reads. We then calculated Q20, Q30, GC content and sequence duplication rate for quality evaluation. All subsequent analyses relied on the obtained clean data. The clean reads were aligned to the reference genome (NCBI accession: JBVYIC000000000) via bwa-mem2 (v2.2, parameters: mem -t 4 -M).

### 2.4. Variant Calling and Annotation

SNP and small insertion/deletion (small InDel) variants were primarily identified using the Genome Analysis Toolkit (GATK) (v3.8, parameters: -XX:ParallelGCThreads = 5 -Xmx20G -T HaplotypeCaller --indelSizeToEliminateInRefModel 50 --emitRefConfidence GVCF --variant_index_type LINEAR --variant_index_parameter 128000) [14]. Briefly, clean read alignments were sorted and deduplicated using Samtools (v1.9). The HaplotypeCaller algorithm in GATK (v3.8) was then employed to generate genomic variant call format (gVCF) files for each sample, followed by joint genotyping across the population. Finally, hard filtering was performed using the criteria QD < 2.0, MQ < 40.0, FS > 60.0, QUAL < 30.0, MQRankSum < −12.5, ReadPosRankSum < −8.0, clusterSize = 2, and clusterWindowSize = 5, yielding high-quality SNP and InDel datasets. Additionally, variants within 5 bp flanking InDels and adjacent InDels within 10 bp were removed using bcftools vcfutils.pl (varFilter -w 5 -W 10). All other filtering parameters followed the official default settings of GATK. Based on the physical positions of the detected variants, functional annotation of SNPs and InDels was performed using SnpEff (v3.6c, default parameters) [15]. SNPs were categorized into intergenic, upstream, downstream, exonic, and intronic regions. Coding-region SNPs were further classified as synonymous or nonsynonymous, while coding-region InDels were assessed for frameshift mutations. For downstream population genetic analyses, SNPs were further filtered to retain only biallelic markers with a minor allele frequency (MAF) ≥ 0.05 and a call rate ≥ 0.80 (i.e., missing rate ≤ 0.20) using VCFtools (v0.1.15, parameters: --maf 0.05 --max-missing 0.20).

### 2.5. Genetic Structure and Genetic Diversity Analysis

Population genetic analyses were performed on 140 individuals using filtered high-quality SNP markers. The phylogenetic tree was constructed with MEGA X [16] using the neighbor-joining method and the Kimura 2-parameter model, with 1000 bootstrap replicates. Population structure was analyzed using Admixture (v1.22, parameters: -C 0.01 -s time --cv -j4) [17] by setting K values from 1 to 10. The optimal number of genetic groups was determined based on cross-validation error. Principal component analysis (PCA) was conducted using EIGENSOFT (v6.0, default parameters) [18]. Selective sweeps were identified with VCFtools (0.1.15, parameters: --fst-window-size 100,000 --fst-window-step 10000 for Fst; --window-pi 100,000 --window-pi-step 10,000 for π) [19] by calculating nucleotide diversity (π) and Fixation Index (Fst) values in 100 kb windows with a 10 kb step size. Genetic diversity was assessed by estimating observed heterozygosity (Ho), expected heterozygosity (He), and nucleotide diversity (π). Additionally, runs of homozygosity (ROH) were identified using the R package detectRUNS with the following parameters: windowSize = 50, threshold = 0.05, minSNP = 50, ROHet = FALSE, maxOppWindow = 1, maxMissWindow = 5, maxGap = 100,000 (bp), minLengthBps = 300,000 (bp), and minDensity = 1/50,000 (i.e., at least one SNP per 50 kb). The individual inbreeding coefficient (F_ROH_) was calculated as the ratio of the total ROH length to the total length of the autosomes.

### 2.6. Gene Flow Analysis

Gene flow analysis was performed using Treemix (v1.13, parameters: -k 1000 -global -m 0 1 2 3 4 5) to visualize interpopulation gene flow among the 140 *A. yunnanensis* samples. To account for linkage disequilibrium, a window size of 1000 (−k) was applied, and the “−global” option was selected to generate the maximum-likelihood tree. Migration events (−m 0 1 2 3 4 5) were sequentially incorporated into the tree to evaluate different gene flow scenarios.

### 2.7. Population Historical Dynamics and Effective Population Size Analysis

We reconstructed the historical effective population size (Ne) dynamics using high-quality SNP data. Coalescent models for each population were built with SMC++ [20]. For each chromosome, the VCF file was converted to SMC format using the command smc++ vcf2smc --ignore-missing -c 10,000 --cores 10 --length [chromosome length]. The --ignore-missing parameter excludes all sites with missing genotypes from the analysis, while the -c 10,000 parameter treats homozygous segments longer than 10,000 bp as missing data to reduce bias from linked sites. According to the evolutionary features of this species, the point mutation rate of the nuclear genome was set to 2.46 × 10^−9^ [21], and the generation length was defined as 2 years. Coalescent simulations based on SNP genotypes were conducted to infer long-term changes in effective population size over historical periods, and the demographic curves of each population were finally visualized.

## 3. Results

### 3.1. Data Quality Control

A total of 1503.03 Gbp of clean data were obtained in this study. The average Q30 value reached 98.20% (Table S1), the average mapping rate against the reference genome was 99.71%, the average sequencing depth was 10×, and the genome coverage was 96.23% (Table S2). The GC content of each sample ranged from 37.26% to 38.34%.

### 3.2. Variant Identification and Annotation

A total of 242,504,293 high-quality SNP loci were identified in this study. After filtering by minor allele frequency (MAF ≥ 0.05) and locus integrity (INT ≥ 0.8, i.e., call rate ≥ 80%), a total of 3,588,793 high-confidence SNP loci were retained for downstream population genetic analyses (Table 2). SNPs were predominantly enriched in intronic regions, with a total of 4,005,120 loci, followed by intergenic regions (2,498,097 loci), upstream regulatory regions (531,406 loci), downstream regulatory regions (514,374 loci), and exonic regions (301,861 loci). A small number of SNPs were distributed in functional regions such as splice sites (Table 3). Within coding regions, non-synonymous mutations (171,069 loci) and synonymous mutations (119,519 loci) constituted the main variant types, accompanied by a small number of functional variants including altered start/stop codons.

**Figure 2:**
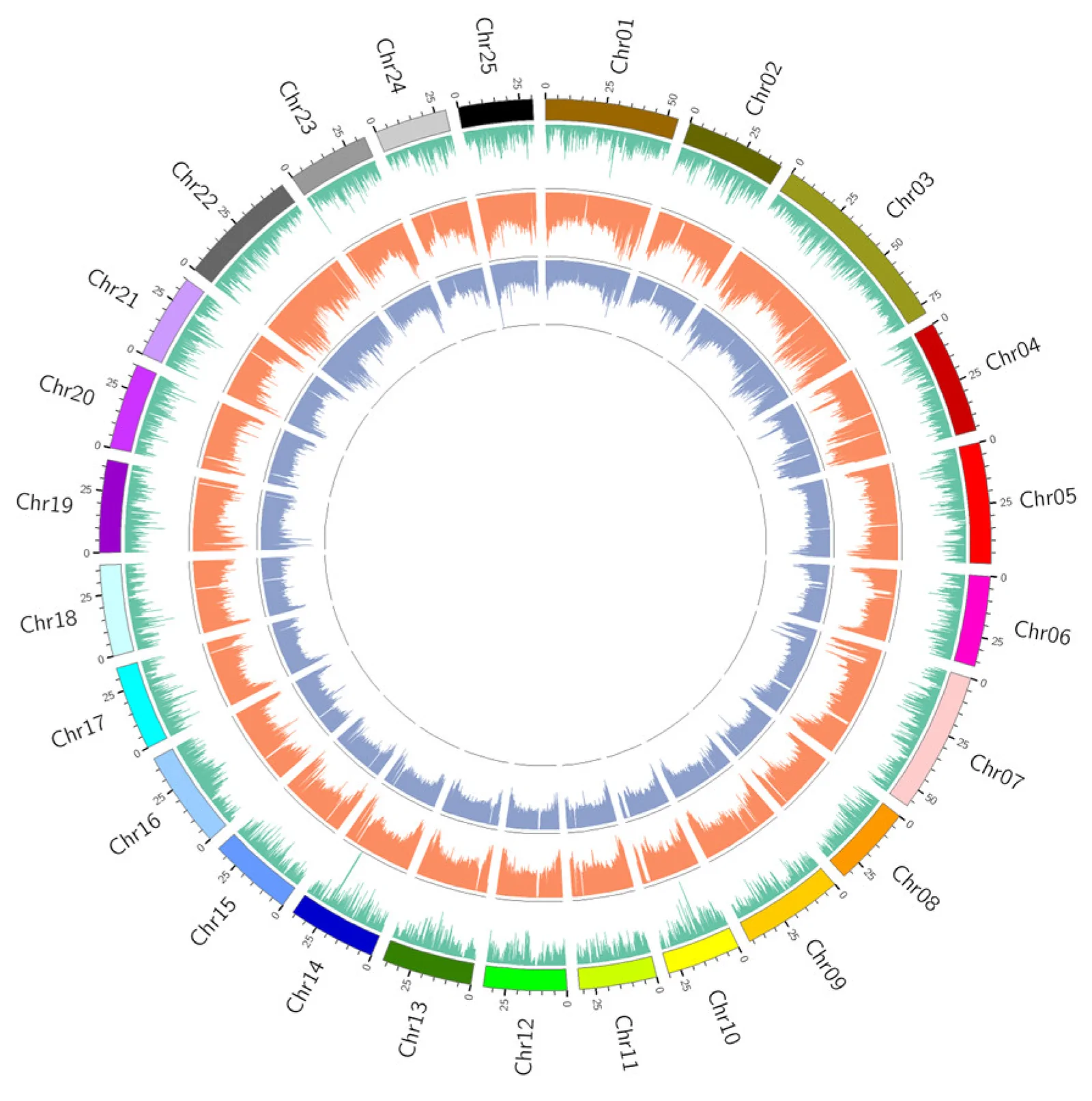
Chromosomal distribution of genetic variations. Tracks from the outermost to the innermost represent chromosomal coordinates, gene density, SNP density, and InDel density, respectively.

The distribution pattern of InDels was consistent with that of SNPs. Specifically, InDels were predominantly enriched in intronic regions, with 1,422,482 loci, followed by intergenic regions (853,520 loci), upstream regions (183,448 loci), downstream regions (181,618 loci), and exonic regions (60,088 loci), while a small number of InDels were located in splice-related regions (Table 3). For coding-region InDels, frameshift mutations were the most abundant, totaling 47,315, along with other functional variants, including codon insertions/deletions and altered stop codons. To intuitively characterize the genome-wide distribution of genetic variation, a Circos plot (v0.69-9) was constructed to visualize the genomic density of SNPs and InDels across the 24 chromosomes of *A. yunnanensis* (Figure 2). The results indicated that both SNPs and InDels were widely and relatively uniformly distributed across all chromosomes, with no obvious clustered aggregation or large-scale deletion regions detected.

### 3.3. Genetic Diversity and Population Differentiation

The expected heterozygosity (He), nucleotide diversity (π) and observed heterozygosity (Ho) were calculated for the seven populations of *A. yunnanensis* (Table 4). Population A had the highest He and Ho but the lowest π among all populations. The other six populations exhibited relatively low and consistent genetic diversity, with He ranging from 0.303 to 0.306, π ranging from 0.0010318 to 0.0010856, and Ho ranging from 0.290 to 0.328. Genetic differentiation between populations was assessed according to Wright’s Fst criteria: low differentiation (Fst ≤ 0.05), moderate differentiation (0.05 < Fst ≤ 0.15), high differentiation (0.15 < Fst ≤ 0.25), and very high differentiation (Fst > 0.25) (Table 5). Population A showed high genetic differentiation from populations B, C, D, E and K, and moderate differentiation from population H. Population H was moderately differentiated from all populations except K. Low genetic differentiation was detected among populations B, C, D, E and K.

### 3.4. Genetic Structure and Traits

**Figure 3:**
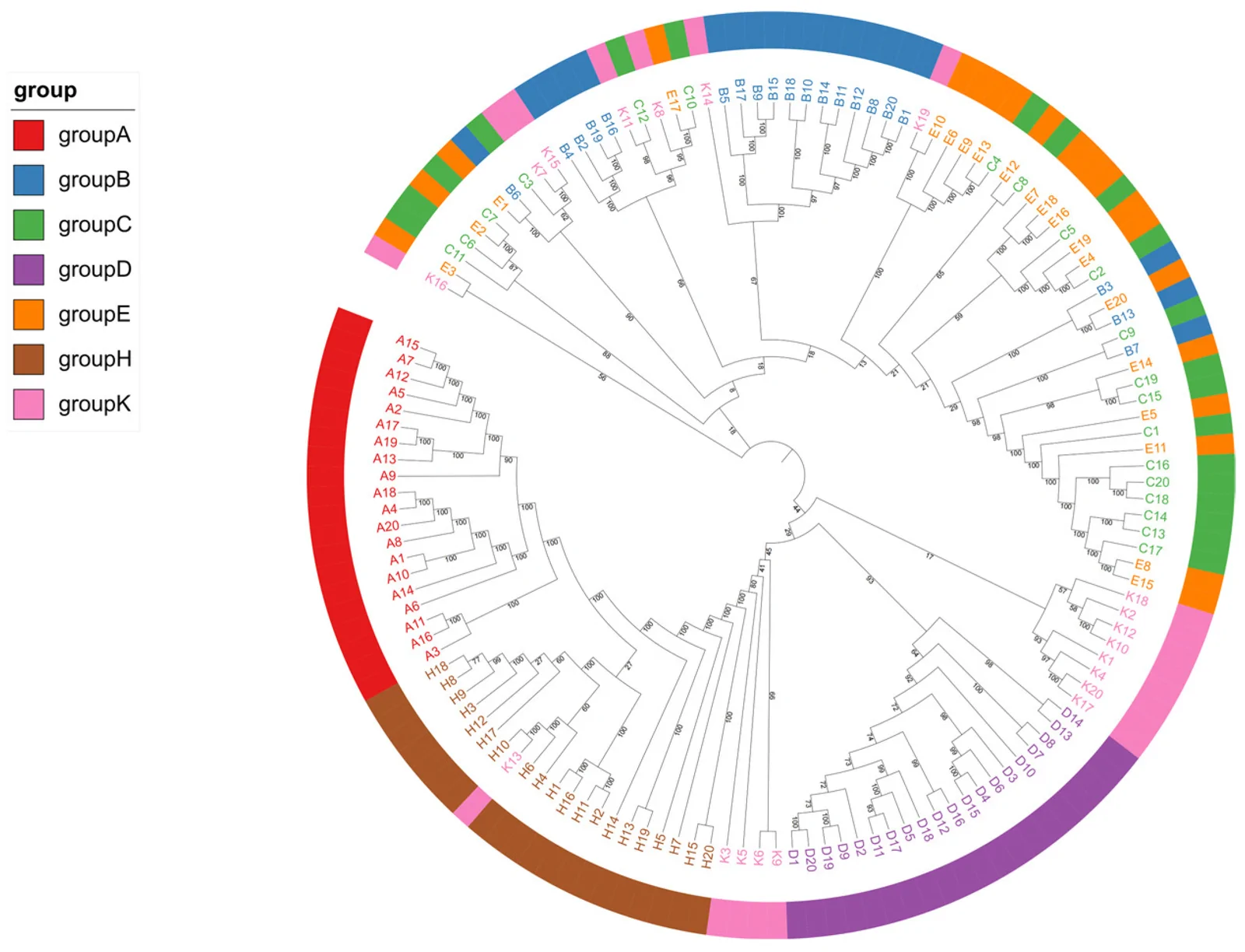
Phylogenetic tree constructed based on SNPs.

The unrooted phylogenetic tree showed obvious genetic differentiation (Figure 3). Population A formed a highly divergent branch that was distinctly separated from all other populations with markedly longer branches. Populations D and H were separated as individual branches, whereas Populations B, C, E and K clustered closely with short internal branches, suggesting weak genetic differentiation among these populations.

**Figure 4:**
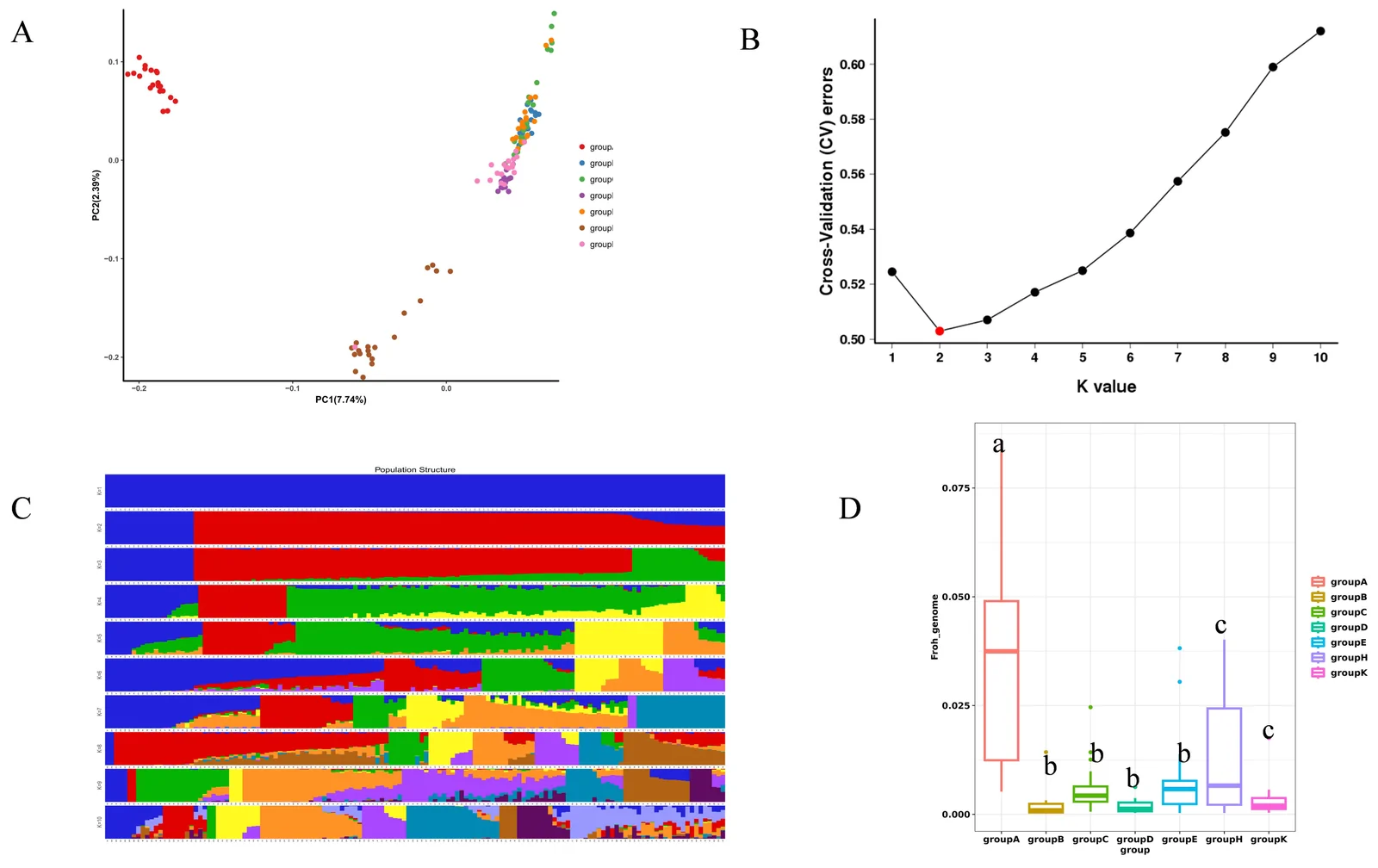
Population genetic structure of seven *A. yunnanensis* populations (**A**) Principal component analysis plot of the seven populations. (**B**) Cross-validation error rates across different K values in Admixture. (**C**) Genetic clustering of individuals under different K values inferred by Admixture. (**D**) Boxplots of genomic inbreeding coefficients (FROH) across seven *A. yunnanensis* populations. Boxes represent the interquartile range (IQR), horizontal lines indicate the medians, and whiskers extend to 1.5× IQR. Different letters above the boxes indicate significant differences (Dunn’s post hoc test, *p* < 0.05).

Principal component analysis (PCA) showed that PC1 and PC2 explained 7.74% and 2.39% of the total genetic variation (Figure 4A), respectively. Individuals from Population A, sampled at the uppermost reach of the Tongche River, were completely separated from the other populations along PC1 and formed an independent genetic cluster. Population H exhibited a relatively distinct distribution along PC2. Samples from populations B, C, D, E and K were highly aggregated, with no obvious separation. Cross-validation was performed across different K values (Figure 4B). The lowest error rate was observed at K = 2, which was determined as the optimal number of genetic clusters. At K = 2, all individuals were divided into two major genetic components (Figure 4C). Population A was almost entirely composed of one component, whereas the other populations were dominated by the second component.

The boxplot intuitively illustrates the distribution and dispersion of F_ROH_ values across the seven *A. yunnanensis* populations. Combined with descriptive statistics including the mean, median, minimum and maximum values, significant statistical differences in F_ROH_ were detected among all groups (Figure 4D). Population A had a mean F_ROH_ of 0.0362 and a median of 0.0375, with values ranging from 0.0052 to 0.0857. It presented the highest median, widest value range and largest data dispersion of all populations. Population H ranked second with a mean F_ROH_ of 0.0126. Populations B, C, D, E and K showed concentrated F_ROH_ distributions, with mean values varying between 0.0018 and 0.0080 and slight numerical discrepancies among these groups (Table 6). Pairwise multiple comparisons demonstrated that Population A differed significantly from every other population. No significant differences were observed among Populations B, C, D and E. Populations H and K were not statistically distinct from each other, yet both differed significantly from B, C, D and E.

**Figure 5:**
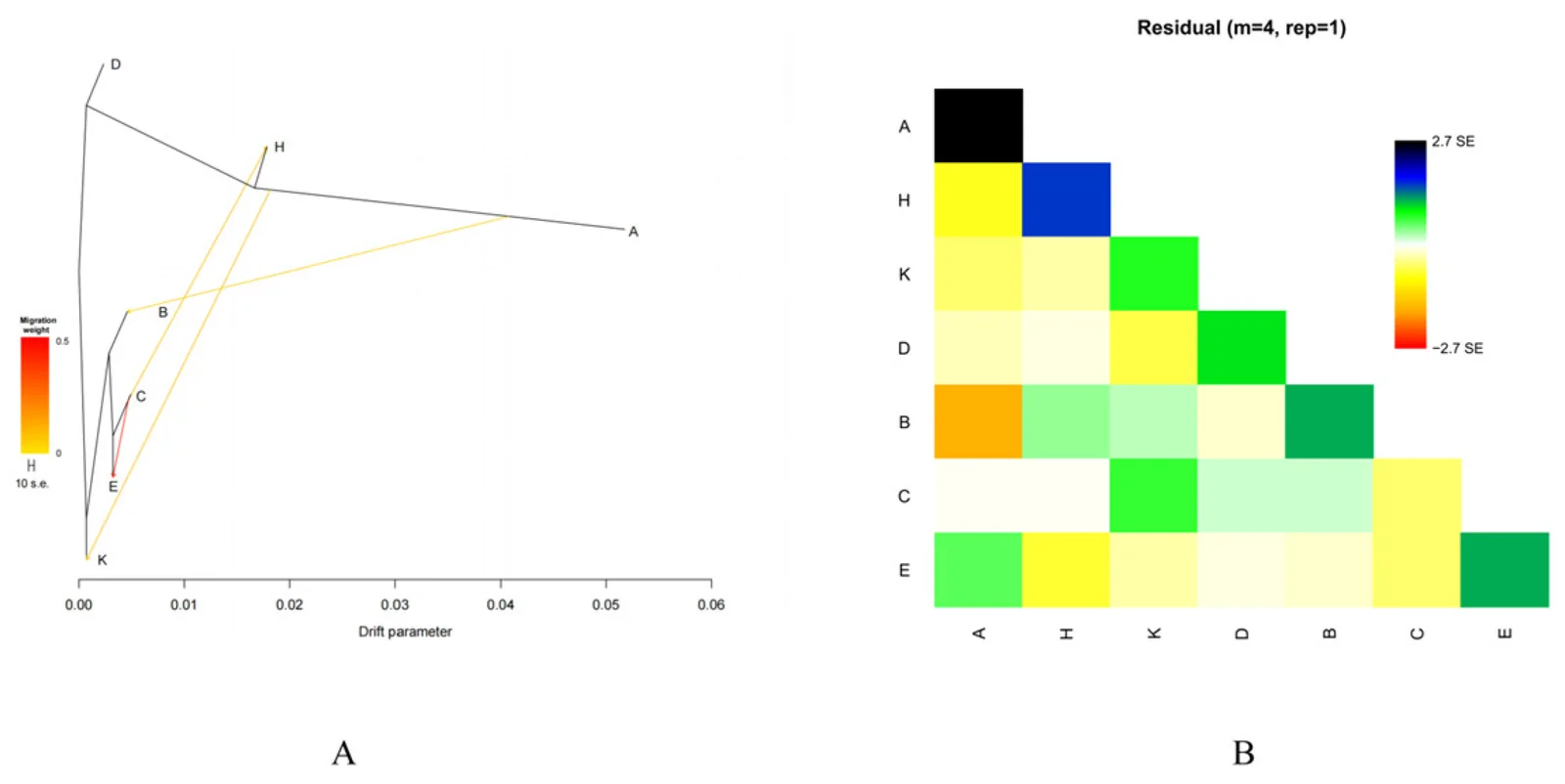
Population phylogeny and gene flow estimated for seven *A. yunnanensis* populations, plotted using TreeMix. (**A**) Population divergence patterns are shown by black branches, and yellow lines indicate the direction of inferred historical gene flow. The color bar on the left denotes migration weights ranging from 0 to 0.5. (**B**) Residual fitting heatmap derived from the maximum-likelihood tree in panel A, with the color scale on the right. White cells correspond to residuals near zero, whereas cells deviating from white reflect fitting bias in pairwise population covariance.

TreeMix analysis was performed to construct a maximum-likelihood population tree and model historical migration events among populations by incorporating migration edges. We determined that the model achieved optimal fitting performance at m = 4, with over 99% of total genetic variance explained by this parameter setting (Figure S1). Four gene flow events were detected under this optimal m value, including gene flow from Population A to B, C to H, A to K, and C to E (Figure 5A). Residual fitting of the model indicated a general signature of historical gene flow across populations. Populations C and E exhibited the closest genetic relatedness, with the strongest gene flow detected between the two groups (Figure 5B).

### 3.5. Population Historical Dynamics

**Figure 6:**
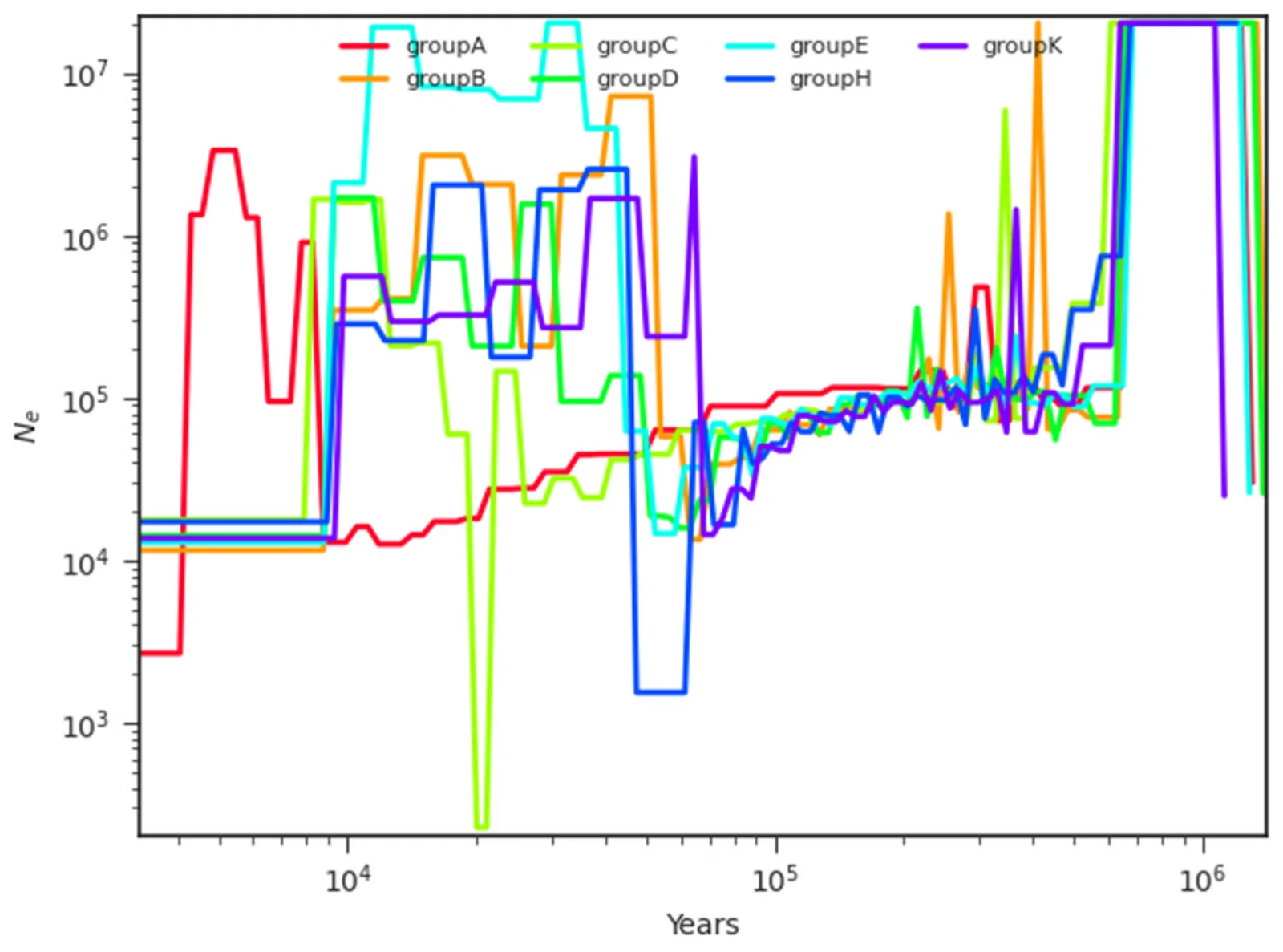
Historical dynamics of effective population size (*Ne*) for seven geographical populations of *A. yunnanensis.*

The effective population size (*Ne*) trajectories of all populations showed broadly similar trends in the early period (Figure 6). During the subsequent prolonged historical stage, the other populations underwent dramatic fluctuations yet maintained relatively large population sizes on the whole. In contrast, Population A was persistently constrained to an extremely low *Ne* across this long timeframe and experienced sustained severe population contraction. However, in the recent historical epoch, the remaining populations, which had fluctuated at high levels for a long time collectively suffered a severe demographic bottleneck, with *Ne* plummeting to a minimum. Meanwhile, Population A, which had persisted at depressed *Ne* values for millennia, underwent rapid demographic expansion. Its Ne surged to an all-time peak over a brief interval before contracting sharply back to an extremely small population size soon thereafter.

### 3.6. Selective Sweep Analysis

**Figure 7:**
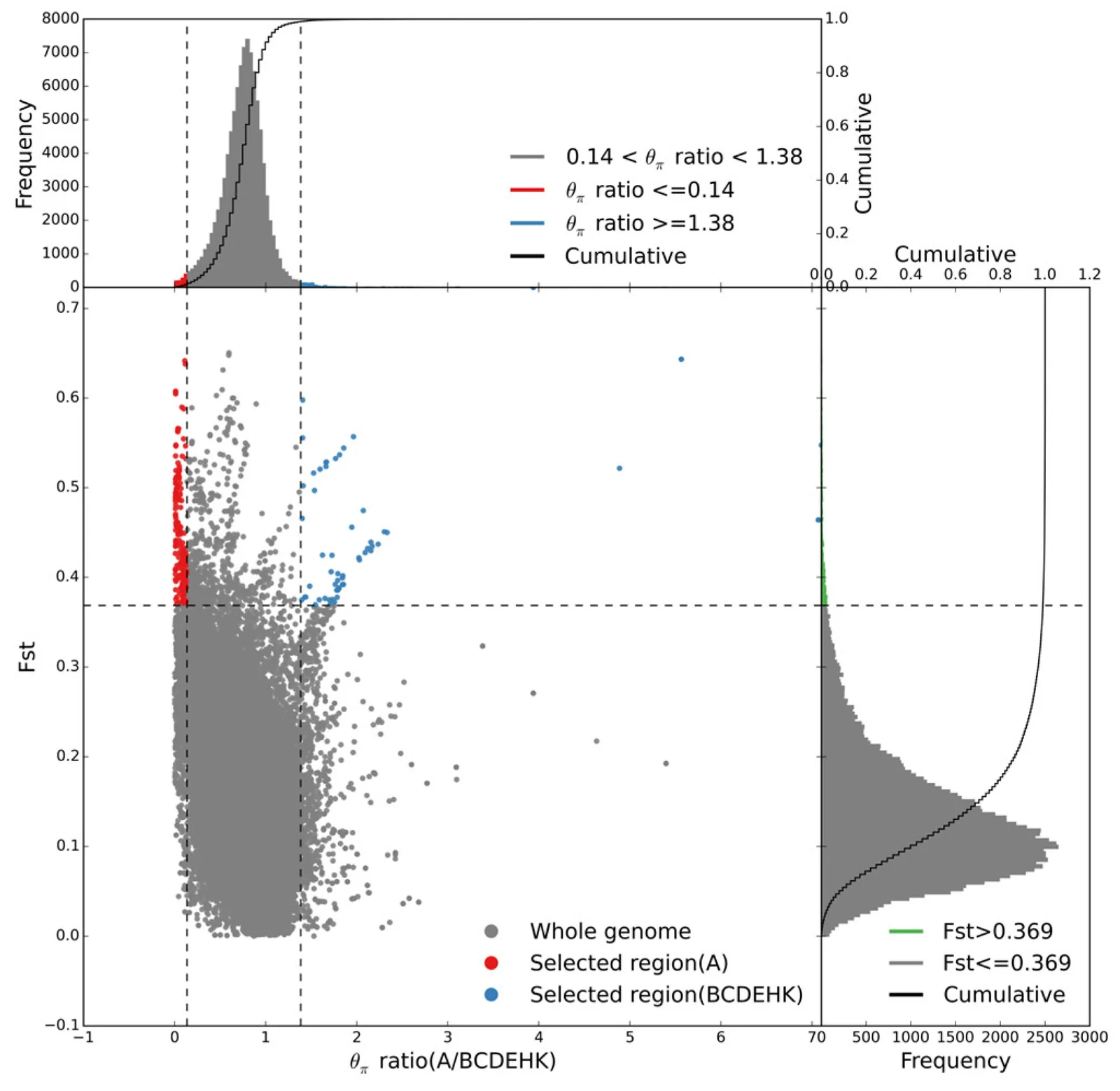
Genome-wide selective sweep scan between *A. yunnanensis* populations based on Fst and θπ ratio. This is a scatter plot of selective sweep analysis based on Fst and θπ ratio. The y-axis represents Fst values, and the x-axis indicates the θπ ratio (θπ ratio = θπA/θπBCDEHK). Gray dots denote genome-wide background loci. Red dots represent candidate selected regions specific to Population A with Fst > 0.369 and θπ ratio ≤ 0.14 (regions with reduced nucleotide diversity in Population A). Blue dots indicate candidate selected regions specific to populations BCDEHK with Fst > 0.369 and θπ ratio ≥ 1.38 (regions with reduced nucleotide diversity in populations BCDEHK). The distribution curves in the upper left and upper right show the frequency and cumulative distributions of θπ ratio and Fst, respectively. All thresholds were defined according to the top 1% quantile of the corresponding indices.

**Figure 8:**
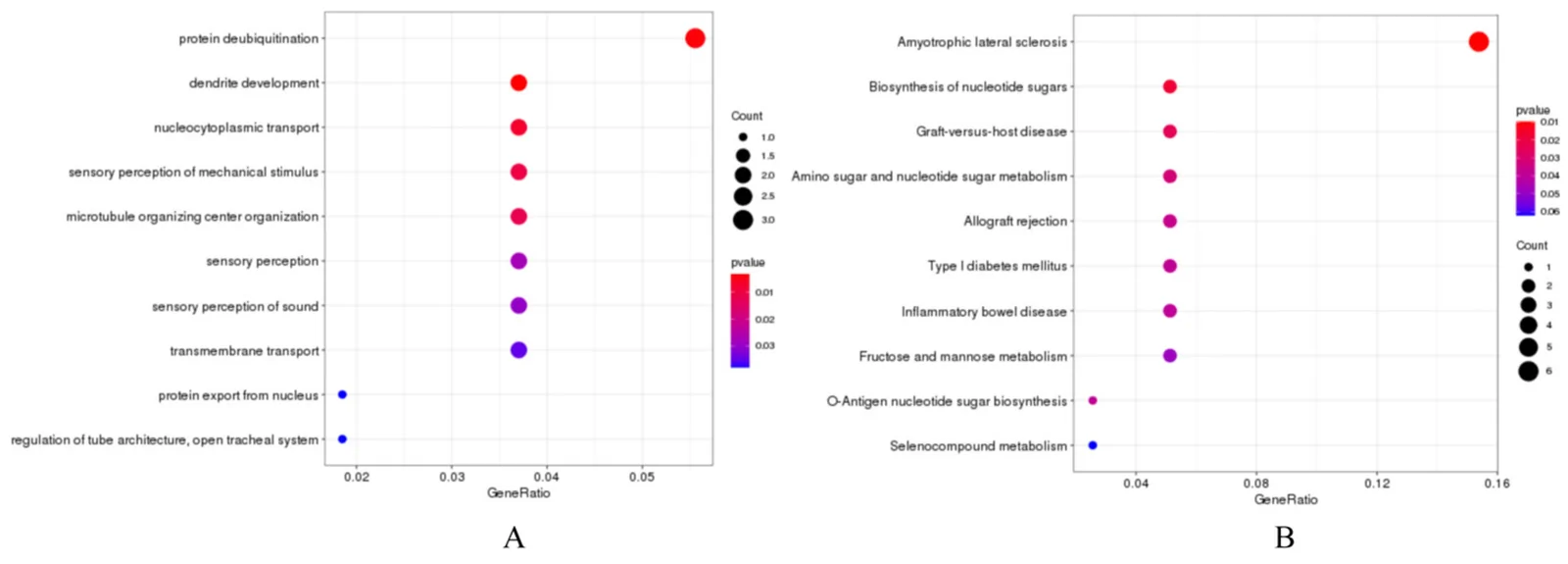
Enrichment analysis of genes within selected regions in Population A. (**A**) GO enrichment analysis. (**B**) KEGG enrichment analysis.

Genome-wide selective sweep scans were performed using the population differentiation index (Fst) and nucleotide diversity ratio (π-ratio). These analyses aimed to detect divergent selection signals. Genomic regions that ranked in the top 1% of both indices were considered candidate regions under selection (Figure 7). Genes located in these regions were then subjected to GO and KEGG enrichment analyses to screen for candidates associated with adaptive differentiation. GO enrichment revealed significant enrichment of candidate genes in functional terms involving mechanosensory perception, dendrite development, microtubule organizing center assembly and protein deubiquitination (Figure 8A). KEGG enrichment demonstrated that these genes were principally enriched in pathways of fructose and mannose metabolism, selenocompound metabolism and amyotrophic lateral sclerosis (Figure 8B). Collectively, ESPN, CROCC, RYK, UNC79, ADPGK, msrB and OTUD7A were pinpointed as core candidate genes underlying the environmental adaptation of Population A.

## 4. Discussion

### 4.1. Genetic Diversity

Genetic diversity is a core indicator for evaluating the evolutionary potential and germplasm health of fish populations, and it directly determines their environmental adaptability and long-term evolutionary capacity [22]. With high-density SNP markers, whole-genome resequencing enables accurate characterization of genetic diversity in wild fish populations. In this study, we analyzed the genetic parameters of seven geographic populations of *A. yunnanensis* from the headwaters of the Chishui River based on genome-wide SNPs. The results showed that the observed heterozygosity (Ho), expected heterozygosity (He) and nucleotide diversity (π) ranged from 0.290 to 0.340, 0.303 to 0.333, and 0.0008063 to 0.0010856 (Table 4), respectively. Zheng and Yang [13] previously reported π values of 0.000182–0.000649 for *A. yunnanensis* using four mitochondrial genes, which were markedly lower than those obtained in the present study. This discrepancy is mainly attributed to the inherent differences in genetic mechanisms between maternally inherited haploid mitochondrial DNA and the nuclear genome. In addition, Jin et al. [23] applied restriction-site associated DNA sequencing (RAD-seq) to the closely related species *Schizothorax lissolabiatus* and obtained slightly lower Ho (0.2381–0.3131) and He (0.2690–0.3115). This pattern may reflect favorable hydrological connectivity in the Chishui River headwaters. Meanwhile, technical bias cannot be ruled out, as RAD-seq has lower detection efficiency for low-frequency variants. Although differences in sequencing platforms and molecular markers hinder direct cross-comparison of absolute values, the whole-genome resequencing used herein, with genome-wide high-density SNPs, provides a more comprehensive and unbiased depiction of the genetic background of wild populations than traditional mitochondrial markers and RAD-seq.

Notably, Population A possessed the highest expected heterozygosity (He = 0.333) and observed heterozygosity (Ho = 0.340) among all seven populations (Table 4), but the lowest nucleotide diversity (π = 0.0008063). As documented by Allendorf et al. (1986), nucleotide diversity is more sensitive to fluctuations in effective population size (Ne) and responds more rapidly to the loss of rare alleles, whereas reductions in heterozygosity occur with an evident time lag; allelic variants are eliminated far more rapidly than the decline rate of heterozygosity during historical population bottlenecks [24,25]. Demographic reconstructions suggested that Population A underwent a prolonged period of continuous *Ne* contraction (Figure 6), while the other populations experienced long-term demographic fluctuations at relatively larger sizes. This prolonged contraction may have induced the permanent loss of numerous rare alleles, consequently reducing genomic π values. Subsequently, this population appeared to experience an abrupt *Ne* expansion followed by repeated demographic oscillations. Although its final Ne stabilized at a relatively low level, most population declines rebounded rapidly and did not develop into severe long-term bottlenecks. Under this scenario, genetic drift may not have eliminated extant alleles extensively, which could account for the persistently high heterozygosity observed in Population A. Neutrality tests, including Tajima‘s D and Fu’s Fs, were excluded from our analyses, as these metrics cannot reliably separate successive demographic contraction and expansion events, whose opposing signatures tend to cancel each other out [26,27]. By contrast, the SMC++ algorithm enables continuous estimation of temporal Ne shifts and is better suited for capturing complicated evolutionary histories [20,28]. We therefore interpret the demographic patterns of Population A based on SMC++ outputs as a robust analytical strategy, while acknowledging that the inferred *Ne* trajectories should be viewed qualitatively rather than as absolute estimates.

Runs of homozygosity (ROH) refer to continuous homozygous segments across the genome, which generally arise when both parents share a common ancestral haplotype. The *F_ROH_* index is defined as the ratio of the total length of ROH to the total genome length, which reflects the individual genomic homozygosity level and is widely used to evaluate the inbreeding degree and genetic diversity of populations. In this study, *F_ROH_* values of seven wild populations were estimated based on ROH analysis, with average values ranging from 0.0015 to 0.0350 (Table 6). Compared with previously reported wild fish populations, the *F_ROH_* values obtained in the present study fell within a relatively low range, indicating low inbreeding levels and favorable genetic diversity backgrounds for most populations [29,30]. Among all groups, the mean *F_ROH_* of Group A (0.035) was significantly higher than those of other populations (Kruskal–Wallis test, *p* < 0.001), accompanied by the largest inter-individual variation (range: 0.005–0.086). Three individuals exhibited *F_ROH_* values exceeding 0.06, suggesting moderate accumulation of inbreeding within this wild population. This pattern may be attributed to its relatively enclosed aquatic habitat, restricted gene flow with the other populations, or historical natural bottleneck events. The average *F_ROH_* values of the remaining six populations were all below 0.013, indicating a low risk of inbreeding. It should be noted that *F_ROH_* estimation is sensitive to detection parameters; thus, the absolute *F_ROH_* values from this study are not suitable for direct numerical comparison with other published datasets, whereas relative differences among populations remain informative.

### 4.2. Genetic Structure

Elucidating population genetic structure can reveal genetic variation and geographic relatedness among populations, and also provide theoretical support for the conservation and scientific management of wild fish germplasm [31]. According to Wright’s criteria [32], Fst < 0.05 indicates low genetic differentiation, 0.05–0.15 indicates moderate differentiation, and 0.15–0.25 indicates high differentiation. In this study, Population A from the upper reaches of the Tongche River presented high genetic differentiation from all other populations, with Fst values ranging from 0.152 to 0.171 (Table 5). Population H in the lower reaches was moderately differentiated from populations B, C, D, E and K (Fst = 0.053–0.062). By contrast, only low differentiation was detected among B, C, D, E and K (Fst = 0.012–0.027). Both phylogenetic reconstruction and population structure analysis divided the seven populations into two major genetic lineages, and PCA further verified that Population A formed a completely independent cluster along the PC1 axis. Collectively, geographical barriers, hydrological connectivity and geographical distance within the river basin are the key drivers of this genetic pattern. Population A inhabits the high-altitude headwater area, where natural barriers such as waterfalls and steep drops exist between upstream and downstream reaches. Long-term geographical isolation has intensified genetic drift and facilitated the formation of a distinct lineage. Previous studies have demonstrated that mountain waterfalls and rapids can effectively block gene flow among stream-dwelling fish and promote the differentiation of native species into independent lineages [33,34,35,36]. The well-connected water systems in the middle and lower reaches allow frequent gene flow among populations B, C, D, E and K, which counteracts genetic drift and results in low differentiation and genetic homogenization [37,38].

We further observed that the pairwise genetic differentiation between Populations H and A was moderate (Fst = 0.1199), distinctly lower than the Fst values (>0.15) between Population A and all remaining populations. Results from phylogenetic reconstruction and PCA consistently showed that Population H deviated along the PC2 axis and occupied an intermediate position between two major genetic populations (Figure 4A). Admixture analysis further uncovered that individuals of Population H carried approximately 36% ancestral components from Population A and 64% ancestral components from the BCDEK lineage, indicating prominent admixed genomic signatures (Figure 4C). Collectively, these lines of evidence suggest that Population H may serve as a potential transitional contact zone mediating genetic exchange between Population A and other conspecific populations. Comparable patterns have been widely documented in freshwater fish studies focusing on secondary contact and genetic introgression. For instance, genetic surveys on *Austrolebias* killifish hybrid zones in South America identified transitional populations with admixed genetic architecture within narrow contact zones between divergent lineages [39]. Research on ricefishes endemic to the ancient lakes of Sulawesi also confirmed persistent interspecific introgression among sympatric congeners, generating populations with intermediate genomic composition [40]. Nevertheless, the optimal TreeMix model detected only unidirectional gene flow from Population C to H, with no direct migration signal from A to H recovered (Figure 5A). This observation does not rule out the transitional population hypothesis for H. Admixed transitional genomes can form via episodic historical population contact and multi-stage complex genetic exchange, rather than sustained, stable unidirectional migration. In addition, TreeMix exhibits limited sensitivity to weak, recent gene flow signals with low migration weights and often fails to capture faint ancient introgression footprints. The detected C to H gene flow also supports stable genetic connectivity between H and the downstream lineages, while the ancestral admixture between A and H likely occurred in the distant past and could not be fully captured by the present model [41]. Given the limitations of the gene flow signals recovered by TreeMix, this hypothesis requires further verification via fine-scale landscape genetics or coalescent simulations [42,43].

### 4.3. Genome-Wide Scan for Selective Sweeps

Selective sweep screening combined with functional annotation identified seven candidate genes (*ESPN, CROCC, RYK, UNC79, ADPGK, msrB,* and *OTUD7A*) that may potentially contribute to the putative local adaptation of the Banbanqiao (A) population to the torrential headwater habitat of the Chishui River (Figure 7 and Figure 8). Robust mechanosensory structures and coordinated neural regulation are essential for survival under rapid flow conditions. *ESPN* encodes an actin-binding protein of inner ear hair cells and lateral line stereocilia, serving as a core component of mechanotransduction. Mammalian studies have shown that overexpression of *Espin* in mice promotes stereociliary development and mitigates its damage, whereas conditional knockout of *Espnl* leads to the fusion and loss of stereocilia [44,45]. Sequence variations in *ESPN* in Population A are hypothesized to possibly improve stereociliary mechanosensitivity by enhancing protein stability or actin-binding efficiency, which could allow individuals to precisely perceive hydrodynamic signals. Functionally coordinated with *ESPN, CROCC* produces a ciliary rootlet protein that anchors sensory ciliary basal bodies to the cytoskeleton. Non-synonymous mutations of crocc2 in cichlid fishes are tightly linked to adaptive craniofacial divergence and phenotypic plasticity, enabling modified mechanical responsiveness of bone tissues [46]. Variants in *CROCC* might strengthen ciliary anchorage against flow shear stress and refine hydrodynamic perception, which may help preserve the intact structure and function of the lateral line system. Integration of sensory inputs and maintenance of a stable locomotor rhythm are prerequisites for rheotactic swimming. Mutations in *RYK*, which encodes an atypical Wnt receptor controlling axon guidance, cause defective hearing and balance function [47]. Similarly, loss of *UNC79* function, which modulates the neuronal resting membrane potential, reduced swimming speed in nematodes by ~60% and disrupted locomotor periodicity [48,49]. We speculate that variants in *RYK* and *UNC79* could optimize the connectivity of sensorimotor circuits and stabilize the baseline excitability of motor neurons, which may improve responsiveness and sustained upstream swimming capacity in turbulent flows.

Beyond neural and sensory adaptation, optimized energy metabolism, antioxidant defense, and proteostasis constitute critical physiological safeguards against exhaustive exercise in Population A. *ADPGK* is an atypical glucokinase that uses *ADP* rather than ATP as a phosphate donor to initiate *ADP*-dependent glycolysis under energy stress. In zebrafish, knockdown of this gene led to severe energy depletion and embryonic lethality [50]. Genetic variants in *ADPGK* in Population A may facilitate anaerobic glycolysis for rapid energy production to support sustained strenuous locomotion. Intensive exercise generates excessive reactive oxygen species (ROS), while the selenoprotein *msrB* specifically repairs oxidized methionine residues. Impaired selenoprotein synthesis in zebrafish triggered marked accumulation of oxidized proteins and developmental malformations [51]. Variants in *msrB* in Population A may enhance ROS elimination and damaged protein restoration, potentially cooperating with *ADPGK* to maintain metabolic homeostasis during high-intensity swimming. The deubiquitinase *OTUD7A* stabilizes cellular homeostasis by inhibiting substrate proteolysis; its knockdown in zebrafish induced morphological abnormalities in the brain, neural tube, and somites [52,53]. Under the combined stresses of rapid flow, hypoxia, and oxidative damage, *OTUD7A* variants could enhance deubiquitinating activity to stabilize rate-limiting proteins involved in mechanosensation, neural conduction, and energy metabolism, which may confer systemic physiological resilience to multiple environmental pressures.

In summary, *ESPN* and *CROCC* may strengthen mechanosensory performance; *RYK* and *UNC79* might optimize sensorimotor coordination; and *ADPGK*, *msrB*, together with *OTUD7A*, could synergistically sustain energy metabolism and proteostasis. Coordinated variation in these functionally distinct genes may form a putative molecular basis for potential local adaptation of Population A to the torrential headwater environment of the Chishui River. Although the biological functions of these genes have been validated in other model organisms, their exact physiological roles and adaptive effects in *A*. *yunnanensis* remain unclear and require further targeted functional verification at the cellular or individual level.

### 4.4. Identification of Management Units and Perspectives

Traditional delineation of Evolutionarily Significant Units (ESUs) and Management Units (MUs) had mostly relied on neutral markers such as mitochondrial sequences, which carry obvious limitations [54]. Modern conservation genomics advocates delineating conservation units by combining neutral lineage divergence and adaptive variation [55]. Based on multi-dimensional genome-wide SNP data, this study conducted a preliminary delineation of conservation units for *A*. *yunnanensis* in the headwaters of the Chishui River. Pairwise Fst values between Population A and all other populations exceeded the high-differentiation threshold of 0.15 (Table 5). Seven functional genes unique to Population A (*ESPN, CROCC, RYK, UNC79, ADPGK, MSRB, OTUD7A*) were identified via genome-wide selective sweep scans (Figure 8); these genes were enriched in pathways related to current-flow adaptation including sensory perception, neural regulation and energy metabolism. TreeMix analyses revealed generally low migration weights toward Population A. Combined with low Nm values, long-term small effective population sizes reconstructed by SMC++ (Figure 6), and elevated F_ROH_ inbreeding coefficients, these lines of evidence collectively indicate long-term restricted genetic exchange between Population A and the remaining populations (Table 6). Integrating neutral genetic divergence, unique adaptive variants, limited gene flow and distinct demographic history, Population A can be tentatively designated as an independent evolutionarily significant unit (ESU), with priority given to habitat conservation, anthropogenic disturbance regulation and long-term genetic monitoring. Notably, a subset of individuals within this population carries clear signatures of inbreeding risk; intensified regular genetic screening and population surveillance should be implemented to track its genomic health status sustainably. Populations B, C, D, E and K from the middle and lower reaches exhibited weak genetic differentiation and frequent gene flow, and consistent signals of genetic homogenization were recovered from phylogenetic reconstruction, PCA and Admixture analyses, supporting their classification as a single MU. Population H harbored approximately 36% ancestral components derived from Population A and occupied an intermediate genetic position between the two major populations (Figure 4). Nevertheless, TreeMix detected only unidirectional gene flow from C to H, with no migration signal from A to H identified, meaning that its transitional status cannot be fully confirmed at present. Given its higher genetic similarity to the downstream populations, Population H was temporarily assigned to the same MU in this study, and further validation through landscape genetics and coalescent simulations is required. Conservation efforts for this population should focus on sustaining hydrological connectivity and mitigating habitat fragmentation. In summary, the classification framework integrating neutral divergence, adaptive variation and population demographic history established in this study provides genomic evidence for the preliminary refined conservation of *A. yunnanensis* germplasm resources.

This study adopted standardized analytical pipelines and SNP filtering parameters that are well established and widely applied in aquatic population genomics, yielding stable and reliable overall results. Nevertheless, varying SNP filtering thresholds may introduce moderate estimation biases into a small number of population genetic parameters during high-throughput genomic analyses. Meanwhile, although this work systematically characterized the genetic diversity and spatial population divergence patterns of *A*. *yunnanensis* in the Chishui River headwaters and identified multiple candidate genes related to local adaptive evolution, long-term continuous fixed-site hydrological and environmental monitoring data are unavailable for this watershed due to difficult mountain field conditions, which prevents quantitative association analyses between genomic variants and field environmental gradients at present. Follow-up research that incorporates long-term field monitoring data including water temperature, flow velocity and dissolved oxygen and combines such data with genomic association analyses can further clarify the intrinsic mechanisms of population divergence driven by environmental selective pressures. Integrating landscape genetics, coalescent simulations and gene functional verification can accurately identify geographic barriers that hinder gene flow within the watershed and reveal the biological functions of adaptive variants. These follow-up investigations are expected to provide more comprehensive theoretical support for the refined conservation of *A*. *yunnanensis* germplasm resources in the headwater reach of the Chishui River.

## 5. Conclusions

Based on whole-genome resequencing, we systematically identified and annotated numerous genetic variants, including SNPs and InDels, across seven *A. yunnanensis* populations from the Chishui River headwaters, and established a genomic variation dataset for these populations. Runs of homozygosity (ROH)-derived inbreeding coefficient (F_ROH_) analysis showed that most populations exhibited generally low genomic inbreeding levels, whereas Population A possessed a relatively higher average F_ROH_ value, with only a small proportion of individuals presenting obvious inbreeding risks. Multiple analyses revealed that Population A underwent independent genetic differentiation, Population H showed moderate divergence, while the remaining geographic populations possessed highly homogeneous genetic backgrounds. Furthermore, Population A has evolved distinct local adaptive traits involving mechanical perception, neuromotor integration, anaerobic metabolic remodeling and protein homeostasis regulation. Our findings provide valuable genomic evidence for germplasm conservation, watershed ecological restoration and the sustainable utilization of *A. yunnanensis* in the headwater region.

## Figures and Tables

**Table 1:** Sampling Sites, Sample IDs, Coordinates and Number of *A. yunnanensis.*

Sampling Sites	Sample ID	Coordinates	Number
Banbanqiao (A)	A1–A20	27.5665° N, 105.1741° E	20
Dashancun (B)	B1–B20	27.7662° N, 105.0729° E	20
Wuqiutian (C)	C1–C20	27.7739° N, 105.1586° E	20
Wanjiaotang (D)	D1–D20	27.7878° N, 105.1575° E	20
Erlongqiangbao (E)	E1–E20	27.7522° N, 105.1808° E	20
Chenjiawan (H)	H1–H20	27.7408° N, 105.2063° E	20
Guohaxia (K)	K1–K20	27.7142° N, 105.2840° E	20

**Table 2:** Summary of SNP filtering steps.

Filtering Step	Filtering Criteria	Remaining SNPs
Raw SNPs	No filter	242,504,293
Post-GATK filter	Quality and cluster cutoffs; remove InDel adjacent sites	7,953,584
Final population filter	MAF and completeness cutoffs; only biallelic SNPs	3,588,793

**Table 3:** Statistics and annotation of SNPs and InDels.

Region/Variant Type	Number of SNPs	Number of InDels
intronic	4,005,120	1,422,482
intergenic	2,498,097	853,520
upstream	531,406	183,448
downstream	514,374	181,618
exonic	301,861	60,088
— synonymous	119,519	—
— nonsynonymous	171,069	—
— frameshift (mutation)	—	47,315
— other coding InDels	—	12,773

**Table 4:** Genetic diversity of the seven *A. yunnanensis* populations.

Group	He	π	Ho
Banbanqiao (A)	0.333	0.0008063	0.340
Dashancun (B)	0.306	0.0010738	0.328
Wuqiutian (C)	0.303	0.0010641	0.290
Wanjiaotang (D)	0.305	0.0010714	0.311
Erlongqiangbao (E)	0.304	0.0010731	0.297
Chenjiawan (H)	0.303	0.0010318	0.307
Guohaxia (K)	0.304	0.0010856	0.300

**Table 5:** Pairwise Fst of the seven *A. yunnanensis* populations.

Group	A	B	C	D	E	H	K
A							
B	0.1713						
C	0.1709	0.0190					
D	0.1617	0.0267	0.0266				
E	0.1666	0.0166	0.0124	0.0232			
H	0.1199	0.0619	0.0604	0.0530	0.0574		
K	0.1522	0.0193	0.0194	0.0163	0.0167	0.0428	

**Table 6:** Inbreeding coefficient (F_ROH_) statistics by population.

Group	Mean	Median	Std	Min	Max
A	0.0362	0.0375	0.0230	0.0052	0.0857
B	0.0022	0.0009	0.0036	0.0003	0.0143
C	0.0060	0.0043	0.0058	0.0006	0.0246
D	0.0018	0.0013	0.0016	0.0003	0.0063
E	0.0080	0.0058	0.0097	0.0003	0.0382
H	0.0126	0.0066	0.0132	0.0003	0.0402
K	0.0031	0.0020	0.0037	0.0004	0.0175

## Data Availability

All sequencing data are deposited in the NCBI BioProject database under accession number PRJNA1478317.

## Supplementary Materials

The following supporting information can be downloaded at https://www.mdpi.com/article/10.3390/ani16142135/s1.
