# Supplementary material for: Population Structure and Local Adaptation of Acrossocheilus yunnanensis in the Headwaters of the Chishui River
Source: Animals (Basel). 2026 Jul 9;16(14):2135. doi: 10.3390/ani16142135 (PMC13404459; doi:10.3390/ani16142135)
Supplement: Supplementary file 1 [file animals-16-02135-s001.zip › animals-4389390-supplementary.pdf]

## Supplementary Tables

**Table S1.** Statistics of Sequencing Data Quality for All Samples.

| Sampies_ID | Clean_Reads | Clean_Base | Q20(%) | Q30(%) | GC(%) |
|---|---|---|---|---|---|
| A1 | 36466434 | 10907725307 | 99.5 | 98.27 | 37.72 |
| A10 | 38220521 | 11430832146 | 99.52 | 98.19 | 38.05 |
| A11 | 38601026 | 11544219409 | 99.52 | 98.22 | 38.19 |
| A12 | 37397278 | 11180309070 | 99.47 | 98.07 | 37.84 |
| A13 | 39372768 | 11774339424 | 99.51 | 98.17 | 38.04 |
| A14 | 34715379 | 10382583667 | 99.46 | 98.02 | 37.99 |
| A15 | 35145257 | 10508715813 | 99.48 | 98.09 | 38.2 |
| A16 | 34698721 | 10374952279 | 99.48 | 98.11 | 38.05 |
| A17 | 37374751 | 11174624809 | 99.55 | 98.31 | 37.96 |
| A18 | 37906145 | 11324816911 | 99.5 | 98.14 | 37.8 |
| A19 | 38252728 | 11442601893 | 99.53 | 98.24 | 37.89 |
| A2 | 34920710 | 10445248885 | 99.52 | 98.27 | 37.68 |
| A20 | 37089702 | 11093840864 | 99.47 | 98.04 | 38.15 |
| A3 | 37645176 | 11260179937 | 99.51 | 98.21 | 37.78 |
| A4 | 35649630 | 10662194879 | 99.54 | 98.31 | 37.78 |
| A5 | 36306726 | 10855414041 | 99.47 | 97.99 | 37.9 |
| A6 | 33688852 | 10077969076 | 99.5 | 98.21 | 37.74 |
| A7 | 37235119 | 11136494733 | 99.49 | 98.13 | 37.99 |
| A8 | 37980122 | 11355696408 | 99.49 | 98.13 | 38.07 |
| A9 | 34209483 | 10228813752 | 99.44 | 97.97 | 37.96 |
| B1 | 36131563 | 10806676793 | 99.54 | 98.32 | 38.03 |
| B10 | 39367634 | 11768342880 | 99.47 | 98.03 | 38.12 |
| B11 | 35642412 | 10658397888 | 99.47 | 98.04 | 38.08 |
| B12 | 34901743 | 10437662238 | 99.49 | 98.12 | 37.8 |
| B13 | 34923142 | 10444735144 | 99.52 | 98.26 | 38.04 |
| B14 | 34860536 | 10424501731 | 99.49 | 98.12 | 37.99 |
| B15 | 37212433 | 11129901628 | 99.53 | 98.26 | 38.1 |
| B16 | 35944358 | 10748981986 | 99.53 | 98.23 | 37.97 |
| B17 | 35909419 | 10739873238 | 99.49 | 98.11 | 37.95 |
| B18 | 35121756 | 10503075179 | 99.5 | 98.13 | 37.93 |
| B19 | 35144423 | 10511052198 | 99.34 | 97.4 | 37.74 |
| B2 | 35826459 | 10715965508 | 99.53 | 98.28 | 37.96 |
| B20 | 34510894 | 10318654750 | 99.48 | 98.1 | 37.89 |
| B3 | 36081183 | 10791681860 | 99.53 | 98.23 | 38.13 |
| B4 | 36303575 | 10858508459 | 99.56 | 98.37 | 37.92 |
| B5 | 35516697 | 10620472697 | 99.48 | 98.08 | 37.81 |
| B6 | 36553848 | 10931850641 | 99.52 | 98.2 | 37.83 |
| B7 | 36540869 | 10924580581 | 99.48 | 98.08 | 38.05 |
| B8 | 38172904 | 11418236115 | 99.55 | 98.3 | 38.03 |
| B9 | 39478753 | 11808728049 | 99.54 | 98.26 | 38.02 |
| C1 | 33658107 | 10063247196 | 99.47 | 98.13 | 38.03 |
| C10 | 36047017 | 10779548730 | 99.52 | 98.22 | 37.9 |
| C11 | 34008559 | 10175624829 | 99.53 | 98.29 | 37.69 |
| C12 | 34462871 | 10310773379 | 99.48 | 98.1 | 37.7 |
| C13 | 37380446 | 11182683912 | 99.52 | 98.2 | 37.73 |
| C14 | 35624423 | 10656922031 | 99.53 | 98.28 | 37.79 |

| | | | | | |
|---|---|---|---|---|---|
| C15 | 35297245 | 10558703522 | 99.5 | 98.15 | 37.74 |
| C16 | 34649502 | 10367096629 | 99.51 | 98.16 | 37.85 |
| C17 | 34690967 | 10380704111 | 99.57 | 98.45 | 37.7 |
| C18 | 35952168 | 10757625213 | 99.55 | 98.37 | 37.83 |
| C19 | 36569533 | 10937690949 | 99.56 | 98.38 | 37.99 |
| C2 | 33659927 | 10054393919 | 99.43 | 97.92 | 37.26 |
| C20 | 34739788 | 10393630779 | 99.48 | 98.11 | 37.85 |
| C3 | 38971406 | 11656164820 | 99.58 | 98.44 | 37.84 |
| C4 | 35106049 | 10502173115 | 99.53 | 98.24 | 38.14 |
| C5 | 34278917 | 10253957861 | 99.55 | 98.29 | 38.07 |
| C6 | 37677492 | 11268218076 | 99.57 | 98.39 | 38.1 |
| C7 | 34964496 | 10455840450 | 99.5 | 98.15 | 38.19 |
| C8 | 39173088 | 11714913154 | 99.51 | 98.13 | 38.08 |
| C9 | 36716443 | 10981311237 | 99.51 | 98.21 | 37.96 |
| D1 | 33718794 | 10089303905 | 99.46 | 97.99 | 37.96 |
| D10 | 35131555 | 10509421163 | 99.36 | 97.39 | 37.69 |
| D11 | 36513656 | 10924916346 | 99.52 | 98.26 | 37.69 |
| D12 | 36736714 | 10990091204 | 99.56 | 98.3 | 37.76 |
| D13 | 37991068 | 11365058911 | 99.57 | 98.4 | 37.6 |
| D14 | 36899510 | 11041948983 | 99.51 | 98.21 | 38.34 |
| D15 | 35572863 | 10642891478 | 99.63 | 98.59 | 37.72 |
| D16 | 36364854 | 10878288387 | 99.44 | 98.05 | 37.63 |
| D17 | 35954044 | 10755846359 | 99.54 | 98.29 | 37.55 |
| D18 | 37419665 | 11196079894 | 99.6 | 98.48 | 37.77 |
| D19 | 34450117 | 10305953551 | 99.56 | 98.37 | 37.82 |
| D2 | 37425329 | 11198269684 | 99.56 | 98.34 | 37.62 |
| D20 | 34615083 | 10354857596 | 99.53 | 98.28 | 37.8 |
| D3 | 36577137 | 10942099302 | 99.52 | 98.2 | 37.89 |
| D4 | 33858315 | 10129117322 | 99.53 | 98.29 | 37.71 |
| D5 | 36877296 | 11032571045 | 99.56 | 98.37 | 37.72 |
| D6 | 35966350 | 10759092296 | 99.51 | 98.17 | 37.81 |
| D7 | 36376035 | 10881725749 | 99.55 | 98.31 | 37.78 |
| D8 | 33959192 | 10157016042 | 99.57 | 98.37 | 37.8 |
| D9 | 35589139 | 10645853753 | 99.5 | 98.17 | 37.75 |
| E1 | 34223630 | 10238825878 | 99.54 | 98.34 | 37.44 |
| E10 | 34770791 | 10398778760 | 99.54 | 98.31 | 37.69 |
| E11 | 34599203 | 10349524008 | 99.55 | 98.36 | 37.76 |
| E12 | 34507143 | 10321076261 | 99.53 | 98.3 | 37.75 |
| E13 | 34141171 | 10212377349 | 99.49 | 98.15 | 37.84 |
| E14 | 34611809 | 10351835779 | 99.52 | 98.24 | 37.71 |
| E15 | 34083732 | 10196759390 | 99.53 | 98.29 | 37.75 |
| E16 | 34137591 | 10211556778 | 99.51 | 98.23 | 37.76 |
| E17 | 38417527 | 11492381318 | 99.48 | 98.05 | 37.7 |
| E18 | 34714931 | 10380046378 | 99.5 | 98.21 | 37.35 |
| E19 | 36529507 | 10928543293 | 99.52 | 98.21 | 37.7 |
| E2 | 35745099 | 10691074640 | 99.48 | 98.1 | 37.62 |
| E20 | 35491778 | 10618013560 | 99.54 | 98.32 | 37.63 |
| E3 | 37271876 | 11151381366 | 99.52 | 98.23 | 37.75 |
| E4 | 36173742 | 10820948279 | 99.49 | 98.11 | 37.81 |
| E5 | 37034721 | 11077292595 | 99.52 | 98.22 | 37.65 |
| E6 | 35144280 | 10516430524 | 99.5 | 98.13 | 37.84 |

| | | | | | |
|---|---|---|---|---|---|
| E7 | 35580998 | 10643261242 | 99.51 | 98.21 | 37.9 |
| E8 | 36243573 | 10842465950 | 99.53 | 98.25 | 37.75 |
| E9 | 35308183 | 10564378818 | 99.55 | 98.37 | 37.72 |
| H1 | 35399288 | 10590439165 | 99.54 | 98.28 | 37.73 |
| H10 | 33875473 | 10132063425 | 99.49 | 98.13 | 37.65 |
| H11 | 36982829 | 11063664211 | 99.56 | 98.34 | 37.74 |
| H12 | 36923535 | 11046228320 | 99.52 | 98.23 | 37.81 |
| H13 | 35926069 | 10746821120 | 99.49 | 98.11 | 37.75 |
| H14 | 39059227 | 11683797707 | 99.46 | 98.08 | 37.61 |
| H15 | 36245139 | 10846026726 | 99.57 | 98.41 | 37.67 |
| H16 | 34524357 | 10327836226 | 99.51 | 98.23 | 37.41 |
| H17 | 34949159 | 10454241914 | 99.52 | 98.22 | 37.65 |
| H18 | 34011828 | 10174623868 | 99.44 | 97.95 | 37.71 |
| H19 | 33397886 | 9987294759 | 99.46 | 98.05 | 37.55 |
| H2 | 37180819 | 11122791593 | 99.56 | 98.37 | 37.76 |
| H20 | 34643055 | 10362532986 | 99.52 | 98.22 | 37.67 |
| H3 | 32178783 | 9624847988 | 99.53 | 98.27 | 37.74 |
| H4 | 36685082 | 10974495815 | 99.52 | 98.23 | 37.72 |
| H5 | 35125211 | 10508331061 | 99.54 | 98.29 | 37.7 |
| H6 | 35178771 | 10523555624 | 99.52 | 98.25 | 37.67 |
| H7 | 34493578 | 10318724336 | 99.53 | 98.26 | 37.75 |
| H8 | 34414793 | 10296148302 | 99.53 | 98.29 | 37.71 |
| H9 | 35366571 | 10577808778 | 99.5 | 98.17 | 37.67 |
| K1 | 35463895 | 10607523198 | 99.5 | 98.14 | 37.69 |
| K10 | 37416597 | 11194457702 | 99.52 | 98.2 | 37.68 |
| K11 | 34453300 | 10306429327 | 99.47 | 98.03 | 37.77 |
| K12 | 37142744 | 11113649434 | 99.54 | 98.28 | 37.66 |
| K13 | 36097855 | 10799491799 | 99.52 | 98.22 | 37.67 |
| K14 | 34789733 | 10406882231 | 99.51 | 98.18 | 37.71 |
| K15 | 35782446 | 10705415172 | 99.58 | 98.48 | 37.69 |
| K16 | 37757597 | 11294737411 | 99.52 | 98.2 | 37.8 |
| K17 | 35413561 | 10592552807 | 99.52 | 98.25 | 37.71 |
| K18 | 39366453 | 11776623951 | 99.55 | 98.31 | 37.75 |
| K19 | 37330480 | 11166727673 | 99.53 | 98.23 | 37.86 |
| K2 | 36308680 | 10861195127 | 99.53 | 98.22 | 37.72 |
| K20 | 36201325 | 10827844186 | 99.52 | 98.21 | 37.3 |
| K3 | 36807107 | 11011204106 | 99.51 | 98.16 | 37.71 |
| K4 | 33857120 | 10127349394 | 99.47 | 98.08 | 37.78 |
| K5 | 37651664 | 11263871190 | 99.49 | 98.07 | 37.44 |
| K6 | 35327180 | 10567574256 | 99.54 | 98.31 | 37.68 |
| K7 | 34553936 | 10334513648 | 99.5 | 98.18 | 37.36 |
| K8 | 34027355 | 10178313781 | 99.52 | 98.24 | 37.59 |
| K9 | 35272639 | 10552466562 | 99.52 | 98.25 | 37.72 |

Notes:Sample_ID refers to the unique identifier for each sample; Clean_Reads represents the number of reads after quality filtering; Clean_Base means the total number of filtered bases, calculated by multiplying the number of clean reads by the read length; Q20(%) indicates the percentage of bases with a Phred quality score of no less than 20 among all clean bases; Q30(%) is the percentage of bases with a Phred quality score of no less than 30; GC(%) stands for the GC content of the sample, which is the proportion of G and C bases in all total bases.

**Table S2.** Mapping Statistics of Sequencing Reads.

| Sample ID | Total_reads | Mapped (%) | Properly_mapped (%) | Ave_depth | Cov_ratio_1X (%) | Cov_ratio_5X (%) | Cov_ratio_10X (%) |
|---|---|---|---|---|---|---|---|
| A1 | 72932868 | 99.75 | 95.1 | 10 | 95.86 | 86.69 | 56.88 |
| A10 | 76441042 | 99.69 | 95.3 | 11 | 96.01 | 87.44 | 59.42 |
| A11 | 77202052 | 99.75 | 95.41 | 11 | 95.59 | 86.75 | 59.06 |
| A12 | 74794556 | 99.63 | 94.24 | 10 | 95.87 | 87.1 | 57.43 |
| A13 | 78745536 | 99.74 | 95.31 | 11 | 96.27 | 87.98 | 61.22 |
| A14 | 69430758 | 99.7 | 95.05 | 10 | 95.67 | 85.12 | 51.81 |
| A15 | 70290514 | 99.77 | 94.84 | 10 | 95.25 | 84.05 | 50.77 |
| A16 | 69397442 | 99.76 | 94.41 | 10 | 95.83 | 84.78 | 50.89 |
| A17 | 74749502 | 99.75 | 94.8 | 10 | 95.74 | 86.31 | 55.81 |
| A18 | 75812290 | 99.73 | 94.8 | 11 | 95.74 | 87.36 | 58.22 |
| A19 | 76505456 | 99.74 | 95.7 | 11 | 95.77 | 87.47 | 60.61 |
| A2 | 69841420 | 99.7 | 95.04 | 10 | 95.77 | 85.93 | 53.18 |
| A20 | 74179404 | 99.73 | 95.09 | 10 | 95.51 | 84.9 | 54.83 |
| A3 | 75290352 | 99.72 | 94.77 | 10 | 95.64 | 87.24 | 59.81 |
| A4 | 71299260 | 99.73 | 94.91 | 10 | 95.69 | 86.33 | 55.4 |
| A5 | 72613452 | 99.73 | 94.39 | 10 | 95.58 | 86.57 | 56.11 |
| A6 | 67377704 | 99.69 | 95.67 | 9 | 95.56 | 84.76 | 50.53 |
| A7 | 74470238 | 99.75 | 95.5 | 10 | 96.31 | 87.08 | 57.76 |
| A8 | 75960244 | 99.74 | 94.97 | 11 | 95.8 | 86.33 | 56.58 |
| A9 | 68418966 | 99.69 | 94.06 | 9 | 95.78 | 84.49 | 49.59 |
| B1 | 72263126 | 99.45 | 95.24 | 10 | 95.88 | 85.09 | 53.09 |
| B10 | 78735268 | 99.72 | 94.19 | 11 | 96.68 | 87.99 | 59.3 |
| B11 | 71284824 | 99.76 | 95.24 | 10 | 96.62 | 86.01 | 53.23 |
| B12 | 69803486 | 99.76 | 95.31 | 10 | 96.82 | 86.53 | 53.16 |
| B13 | 69846284 | 99.72 | 95.01 | 10 | 96.04 | 84.71 | 51.02 |
| B14 | 69721072 | 99.67 | 94.78 | 10 | 97.16 | 85.46 | 51.01 |
| B15 | 74424866 | 99.73 | 95.13 | 10 | 96.81 | 86.52 | 55.07 |
| B16 | 71888716 | 99.76 | 95.78 | 10 | 97.29 | 87.29 | 54.89 |
| B17 | 71818838 | 99.75 | 95.13 | 10 | 96.39 | 86.31 | 54.63 |
| B18 | 70243512 | 99.75 | 95.53 | 10 | 96.63 | 86.43 | 53.4 |
| B19 | 70288846 | 99.77 | 95.64 | 10 | 97.37 | 86.57 | 53.61 |
| B2 | 71652918 | 99.64 | 95.55 | 10 | 97.26 | 86.63 | 54.52 |
| B20 | 69021788 | 99.77 | 95.32 | 10 | 97.05 | 86.01 | 51.82 |
| B3 | 72162366 | 99.76 | 95.08 | 10 | 96.75 | 85.84 | 53.62 |
| B4 | 72607150 | 99.74 | 95.37 | 10 | 96.15 | 86.34 | 55.02 |
| B5 | 71033394 | 99.71 | 95.09 | 10 | 97.01 | 86.66 | 53.96 |
| B6 | 73107696 | 99.75 | 95.06 | 10 | 96.29 | 86.75 | 56.32 |
| B7 | 73081738 | 99.75 | 94.32 | 10 | 96.5 | 86.54 | 54.15 |
| B8 | 76345808 | 99.74 | 95.53 | 11 | 96.46 | 87.58 | 59.38 |
| B9 | 78957506 | 99.78 | 95.37 | 11 | 96.83 | 87.99 | 61.13 |
| C1 | 67316214 | 99.75 | 94.78 | 9 | 96.08 | 84.36 | 48.25 |
| C10 | 72094034 | 99.72 | 94.16 | 10 | 96.23 | 85.47 | 52.87 |
| C11 | 68017118 | 99.69 | 96.36 | 9 | 96.36 | 84.53 | 50.4 |
| C12 | 68925742 | 99.67 | 96.35 | 10 | 95.61 | 82.7 | 50.15 |
| C13 | 74760892 | 99.74 | 96.1 | 10 | 95.88 | 87.13 | 59.44 |
| C14 | 71248846 | 99.71 | 96.02 | 10 | 96.06 | 86.36 | 54.98 |
| C15 | 70594490 | 99.72 | 95.82 | 10 | 96.22 | 86.32 | 54.53 |
| C16 | 69299004 | 99.7 | 95.93 | 10 | 95.85 | 84.41 | 51.57 |
| C17 | 69381934 | 99.68 | 96.55 | 10 | 95.41 | 83.81 | 51.57 |
| C18 | 71904336 | 99.7 | 96.06 | 10 | 95.91 | 84.74 | 53.73 |
| C19 | 73139066 | 99.77 | 95.91 | 10 | 96.47 | 87.13 | 56.56 |
| C2 | 67319854 | 99.71 | 93.35 | 9 | 96.22 | 83.91 | 45.91 |
| C20 | 69479576 | 99.67 | 95.68 | 10 | 95.73 | 83.34 | 50.7 |
| C3 | 77942812 | 99.75 | 94.86 | 11 | 96.75 | 88.6 | 61.43 |
| C4 | 70212098 | 99.76 | 95.85 | 10 | 96 | 84.95 | 52.35 |
| C5 | 68557834 | 99.73 | 94.93 | 10 | 96.19 | 84.07 | 49.35 |
| C6 | 75354984 | 99.76 | 95.22 | 11 | 96.31 | 86.87 | 56.95 |
| C7 | 69928992 | 99.73 | 94.97 | 10 | 96.26 | 84.73 | 50.23 |
| C8 | 78346176 | 99.71 | 94.85 | 11 | 96.55 | 87.89 | 59.82 |
| C9 | 73432886 | 99.73 | 94.98 | 10 | 96.18 | 86.62 | 55.88 |
| D1 | 67437588 | 99.69 | 95.42 | 9 | 96.12 | 83.5 | 48.97 |
| D10 | 70263110 | 99.61 | 95.97 | 10 | 96.24 | 86.01 | 53.85 |

| | | | | | | | |
|---|---|---|---|---|---|---|---|
| D11 | 73027312 | 99.57 | 95.91 | 10 | 96.25 | 85.57 | 55.22 |
| D12 | 73473428 | 99.74 | 96.04 | 10 | 96.28 | 87.3 | 58.13 |
| D13 | 75982136 | 99.77 | 96.33 | 11 | 96.42 | 87.74 | 60.23 |
| D14 | 73799020 | 99.62 | 95.24 | 10 | 96.04 | 84.24 | 53.63 |
| D15 | 71145726 | 99.76 | 96.24 | 10 | 97.01 | 86.33 | 54.65 |
| D16 | 72729708 | 99.73 | 95.92 | 10 | 96.28 | 86.92 | 56.89 |
| D17 | 71908088 | 99.61 | 95.88 | 10 | 96.74 | 86.93 | 56.19 |
| D18 | 74839330 | 99.71 | 96.07 | 10 | 96.49 | 87.53 | 59.25 |
| D19 | 68900234 | 99.72 | 96 | 10 | 96.23 | 85.71 | 52.37 |
| D2 | 74850658 | 99.73 | 96.11 | 10 | 96.35 | 87 | 59.08 |
| D20 | 69230166 | 99.71 | 95.88 | 10 | 96.16 | 85.91 | 52.66 |
| D3 | 73154274 | 99.75 | 95.83 | 10 | 96.04 | 86.63 | 56.9 |
| D4 | 67716630 | 99.74 | 95.92 | 9 | 96.97 | 85.18 | 50.66 |
| D5 | 73754592 | 99.72 | 96.02 | 10 | 96.43 | 87.04 | 58.24 |
| D6 | 71932700 | 99.77 | 96.18 | 10 | 96 | 86.13 | 55.6 |
| D7 | 72752070 | 99.77 | 96.09 | 10 | 96.22 | 86.95 | 57.24 |
| D8 | 67918384 | 99.7 | 95.74 | 9 | 96.57 | 85.53 | 51.12 |
| D9 | 71178278 | 99.76 | 95.84 | 10 | 96.31 | 86.49 | 54.67 |
| E1 | 68447260 | 99.71 | 95.88 | 9 | 95.92 | 85.23 | 51.64 |
| E10 | 69541582 | 99.65 | 95.79 | 10 | 96.2 | 86.1 | 52.73 |
| E11 | 69198406 | 99.7 | 95.87 | 10 | 96.17 | 85.84 | 52.27 |
| E12 | 69014286 | 99.72 | 95.77 | 10 | 96.57 | 85.89 | 52.2 |
| E13 | 68282342 | 99.73 | 96.05 | 9 | 96.1 | 85.53 | 51.26 |
| E14 | 69223618 | 99.74 | 96.19 | 10 | 96.32 | 86.15 | 52.8 |
| E15 | 68167464 | 99.74 | 96.09 | 9 | 96.08 | 85.39 | 51.22 |
| E16 | 68275182 | 99.76 | 96.29 | 9 | 96.49 | 85.93 | 51.57 |
| E17 | 76835054 | 99.72 | 95.9 | 11 | 96.14 | 87.85 | 61.55 |
| E18 | 69429862 | 99.74 | 94.29 | 10 | 96.34 | 86.29 | 52.73 |
| E19 | 73059014 | 99.73 | 95.87 | 10 | 96.36 | 87.22 | 57.46 |
| E2 | 71490198 | 99.73 | 95.28 | 10 | 96.11 | 86.77 | 55.68 |
| E20 | 70983556 | 99.71 | 95.96 | 10 | 96.21 | 86.43 | 55.1 |
| E3 | 74543752 | 99.69 | 95.99 | 10 | 96.17 | 87.34 | 59.38 |
| E4 | 72347484 | 99.72 | 95.97 | 10 | 96.45 | 86.99 | 56.48 |
| E5 | 74069442 | 99.7 | 95.45 | 10 | 96.49 | 87.69 | 58.42 |
| E6 | 70288560 | 99.69 | 95.95 | 10 | 95.75 | 85.04 | 53.41 |
| E7 | 71161996 | 99.76 | 95.9 | 10 | 96.45 | 86.74 | 54.69 |
| E8 | 72487146 | 99.69 | 96.01 | 10 | 96.18 | 86.93 | 56.72 |
| E9 | 70616366 | 99.7 | 96.35 | 10 | 96.17 | 86.04 | 54.03 |
| H1 | 70798576 | 99.66 | 95.8 | 10 | 95.79 | 85.86 | 54.51 |
| H10 | 67750946 | 99.7 | 95.78 | 9 | 95.51 | 84.92 | 51.05 |
| H11 | 73965658 | 99.73 | 95.98 | 10 | 96.42 | 87.07 | 58.35 |
| H12 | 73847070 | 99.72 | 95.73 | 10 | 96.13 | 86.97 | 58.05 |
| H13 | 71852138 | 99.73 | 95.79 | 10 | 96.74 | 86.66 | 56.26 |
| H14 | 78118454 | 99.72 | 95.44 | 11 | 96.73 | 88.33 | 62.78 |
| H15 | 72490278 | 99.7 | 96.26 | 10 | 96.43 | 86.17 | 55.85 |
| H16 | 69048714 | 99.7 | 95.76 | 10 | 96.32 | 85.4 | 52.4 |
| H17 | 69898318 | 99.7 | 95.73 | 10 | 96.14 | 85.91 | 53.72 |
| H18 | 68023656 | 99.72 | 95.77 | 9 | 95.98 | 85.19 | 51.11 |
| H19 | 66795772 | 99.72 | 94.57 | 9 | 96.02 | 85.02 | 49.67 |
| H2 | 74361638 | 99.74 | 96.1 | 10 | 96.02 | 87.15 | 58.84 |
| H20 | 69286110 | 99.7 | 95.67 | 10 | 96.31 | 85.87 | 52.77 |
| H3 | 64357566 | 99.71 | 95.62 | 9 | 95.76 | 83.51 | 46.38 |
| H4 | 73370164 | 99.72 | 95.28 | 10 | 96.25 | 87.08 | 57.69 |
| H5 | 70250422 | 99.72 | 95.87 | 10 | 96.15 | 86.16 | 53.96 |
| H6 | 70357542 | 99.71 | 95.69 | 10 | 95.84 | 85.79 | 54.33 |
| H7 | 68987156 | 99.73 | 95.89 | 10 | 96.2 | 85.88 | 52.44 |
| H8 | 68829586 | 99.67 | 95.77 | 10 | 95.92 | 85.5 | 52.26 |
| H9 | 70733142 | 99.71 | 95.65 | 10 | 95.89 | 86.14 | 54.73 |
| K1 | 70927790 | 99.64 | 95.53 | 10 | 96.56 | 86.65 | 54.86 |
| K10 | 74833194 | 99.69 | 95.93 | 10 | 96.37 | 87.46 | 59.42 |
| K11 | 68906600 | 99.74 | 95.9 | 10 | 96.08 | 85.6 | 52.66 |
| K12 | 74285488 | 99.69 | 96.1 | 10 | 96.16 | 86.18 | 57.1 |
| K13 | 72195710 | 99.71 | 95.88 | 10 | 95.99 | 86.44 | 56.35 |
| K14 | 69579466 | 99.74 | 95.77 | 10 | 96.24 | 86.23 | 53.42 |
| K15 | 71564892 | 99.61 | 95.69 | 10 | 95.86 | 84.91 | 53.69 |
| K16 | 75515194 | 99.72 | 95.94 | 11 | 96.35 | 87.69 | 60.07 |

| | | | | | | | |
|---|---|---|---|---|---|---|---|
| K17 | 70827122 | 99.59 | 95.61 | 10 | 96.04 | 86.37 | 54.98 |
| K18 | 78732906 | 99.49 | 95.5 | 11 | 96.48 | 88.16 | 62.8 |
| K19 | 74660960 | 99.44 | 95.2 | 10 | 96.4 | 87.42 | 58.32 |
| K2 | 72617360 | 99.7 | 95.72 | 10 | 96.23 | 86.83 | 56.9 |
| K20 | 72402650 | 99.73 | 95.61 | 10 | 96.61 | 87 | 56.26 |
| K3 | 73614214 | 99.72 | 95.41 | 10 | 96.55 | 87.41 | 57.76 |
| K4 | 67714240 | 99.74 | 95.79 | 9 | 96.5 | 85.64 | 50.62 |
| K5 | 75303328 | 99.74 | 95.89 | 11 | 96.32 | 87.53 | 59.62 |
| K6 | 70654360 | 99.71 | 95.86 | 10 | 96.37 | 86.48 | 54.35 |
| K7 | 69107872 | 99.75 | 94.99 | 10 | 96.27 | 86.05 | 52.26 |
| K8 | 68054710 | 99.74 | 95.71 | 9 | 96.28 | 85.46 | 51.04 |
| K9 | 70545278 | 99.45 | 95.57 | 10 | 96.3 | 86.24 | 54.01 |

Notes: Sample ID: Unique identifier for each sample, Total_reads: Total number of clean reads used for alignment Mapped (%):Percentage of clean reads successfully mapped to the reference genome , Properly_mapped (%): Percentage of paired-end reads where both ends align to the reference genome with insert sizes consistent with the sequenced fragment library, Ave_depth: Average sequencing depth of the sample, Cov_ratio_X (%): Proportion of bases in the reference genome covered by at least the specified sequencing depth (1×, 5×, and 10×, respectively). For example, Cov_ratio_1X (%) indicates the percentage of bases across the reference genome covered by at least one read.

## Supplementary Figure

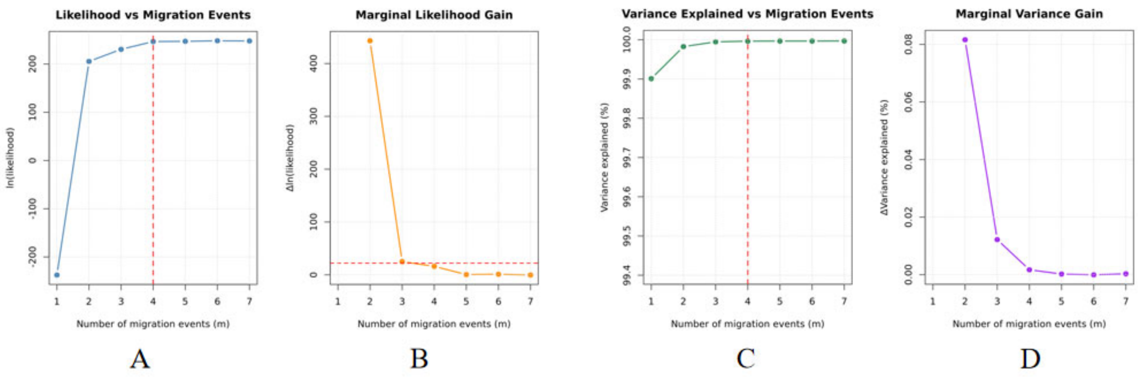


**Figure S1.** Determination of optimal migration edges (m) for TreeMix. (**A**) Log-likelihood across different migration event counts; saturation at m = 4. (**B**) Marginal log-likelihood gain for each added migration edge. (**C**) Percentage of variance explained by TreeMix models with varying m values. (**D**) Marginal gain in explained variance for each migration edge increment.
